# Segmental and site-specific isotope labelling strategies for structural analysis of posttranslationally modified proteins

**DOI:** 10.1039/d1cb00045d

**Published:** 2021-08-11

**Authors:** Dominik P. Vogl, Anne C. Conibear, Christian F. W. Becker

**Affiliations:** University of Vienna, Faculty of Chemistry, Institute of Biological Chemistry Währinger Straße 38 1090 Vienna Austria christian.becker@univie.ac.at +43-1-4277-870510 +43-1-4277-70510; The University of Queensland, School of Biomedical Sciences St Lucia Brisbane 4072 QLD Australia

## Abstract

Posttranslational modifications can alter protein structures, functions and locations, and are important cellular regulatory and signalling mechanisms. Spectroscopic techniques such as nuclear magnetic resonance, infrared and Raman spectroscopy, as well as small-angle scattering, can provide insights into the structural and dynamic effects of protein posttranslational modifications and their impact on interactions with binding partners. However, heterogeneity of modified proteins from natural sources and spectral complexity often hinder analyses, especially for large proteins and macromolecular assemblies. Selective labelling of proteins with stable isotopes can greatly simplify spectra, as one can focus on labelled residues or segments of interest. Employing chemical biology tools for modifying and isotopically labelling proteins with atomic precision provides access to unique protein samples for structural biology and spectroscopy. Here, we review site-specific and segmental isotope labelling methods that are employed in combination with chemical and enzymatic tools to access posttranslationally modified proteins. We discuss illustrative examples in which these methods have been used to facilitate spectroscopic studies of posttranslationally modified proteins, providing new insights into biology.

## Abstract 2

Selective isotope labeling facilitates the analysis of effects of posttranslational modifications on protein structure and function.

## Graphical abstract

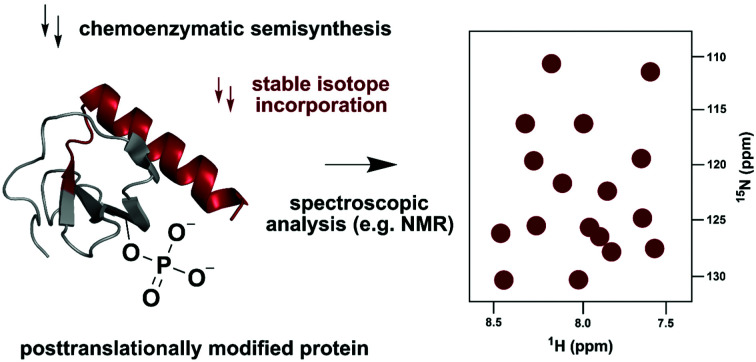

## Introduction

The number of protein variants (proteoforms) in eukaryotes often vastly exceeds the number of protein-coding genes. The main source of this proteoform diversity is posttranslational modification (PTM) of proteins, which can occur on backbone and side-chain functional groups and include addition of functional groups, cyclization, splicing and cleavage.^1^ These PTMs are introduced either enzymatically or non-enzymatically and functional groups added vary considerably in complexity and size.^2^ Nearly all eukaryotic proteins are posttranslationally modified during their lifetime, with diverse effects on protein structure, dynamics and function. The large number of PTMs, their chemical diversity and overall heterogeneity makes the study of their individual effects challenging but also essential to understanding the molecular mechanisms that control life.^3–5^

Understanding the structural effects of PTMs and how these influence protein function, dynamics and interaction with binding partners is enabled by a variety of structural biology techniques. Spectroscopic techniques such as Nuclear Magnetic Resonance (NMR), infrared (IR) and Raman spectroscopy allow for the study of protein structure at atomic resolution and can provide insights into protein dynamics even in highly flexible regions. These spectroscopic techniques combined with X-ray crystallography, which has been most extensively used for protein structure determination, are now complemented by cryo-electron microscopy, which has revolutionised structural biology by enabling structural studies of large complexes at increasing resolution.^6^ Integrated structural biology approaches take advantage of the strengths of multiple techniques and show great promise for elucidating the mechanisms of protein regulation by PTMs.

Spectroscopic techniques applied to understanding the effects of PTMs on protein structures and mechanisms each have inherent advantages and limitations. Biomolecular NMR spectroscopy, for example, can provide information on conformational preferences and changes in flexible regions that are not visible in rigid crystal structures.^7^ PTMs can cause local alterations in the chemical environments of protein nuclei, resulting in characteristic chemical shift changes that can be used to identify the PTM type and site of modification if residue assignments are available.^8^ Solution NMR also offers the possibility to monitor enzymatic introduction of PTMs and their subsequent removal in a quantitative and non-disruptive manner under native conditions.^9^ However, protein size can be a limitation for solution NMR due to long correlation times and spectral complexity for large proteins. Combining NMR with scattering techniques such as SAXS (small-angle X-ray scattering) or SANS (small-angle neutron scattering) opens up the possibility of studying PTM-induced conformational changes even in macromolecular complexes or multidomain proteins.^10^ Vibrational spectroscopy, such as IR and Raman, can deliver insights into covalent bonds in a molecule based on vibrational energy transitions and therefore can report on PTM-induced structural changes (IR) or can provide valuable information about the overall secondary structure (Raman).^11,12^ Other spectroscopic techniques frequently applied to studying proteins (and the effects of PTMs) such as UV/vis- and fluorescence spectroscopy are beyond the scope of this article, as they do not rely on specific isotope labelling patterns.^13^ Mass spectrometry-based proteomics strategies also benefit greatly from isotope labels in certain quantitative applications such as SILAC (stable isotope labelling by/with amino acids in cell culture), however labelling is typically not site-selective or segmental in these applications and therefore not covered here. Please refer to recent reviews on mass spectrometry-based proteomics for further details.^14^

Here we focus on spectroscopic applications that require or greatly benefit from the incorporation of specific stable isotopes at non-natural abundance – namely NMR, IR, Raman and small-angle scattering (SAS) technologies to study defined PTMs in proteins. We provide an overview of the current chemical and enzymatic toolbox for segmental and site-specific isotope labelling strategies for proteins. After summarizing the available methodologies, we highlight their strengths and weaknesses using selected examples of proteins carrying a variety of PTMs.

## Isotope labelling of proteins for spectroscopy

Biomolecular NMR spectroscopy relies on the spin-1/2 of ^1^H, ^13^C and ^15^N nuclei.^15^ Homonuclear ^1^H-NMR takes advantage of the 99.98% natural abundance of ^1^H but suffers from signal overlap for proteins larger than ∼50 residues because of the large number of protons in biological molecules and relatively narrow range of proton resonances.^16^ Multidimensional spectra that correlate protons with nitrogen and carbon in proteins can reduce the number of signals and increase resolution, but are limited by the low natural abundance of NMR-active carbon and nitrogen isotopes (1.1% for ^13^C and 0.366% for ^15^N).^17^ Larger proteins can be deuterated in order to reduce disadvantageous dipolar interactions and adverse spin diffusion.^18^ For example, combining ^2^H–^13^C–^15^N isotope labelling of proteins with transverse relaxation optimized spectroscopy (TROSY), an NMR experiment that reduces the effective transverse relaxation and thereby decreases linewidth and increases sensitivity of signals from large molecules with long correlation times,^19^ larger proteins as well as complexes typically up to 100 kDa in molar mass can be studied by NMR. In certain cases, using deuteration in combination with methyl group labelling, protein assemblies of up to 1 MDa have been successfully studied by NMR.^20^ Alternative NMR-active nuclei that occur at 100% natural abundance, such as ^19^F, are seeing an increasing use in NMR applications (*e.g.* in screening approaches), due to their sensitivity and uncrowded spectra.^21^ However, as they seldom occur naturally in proteins, we do not discuss incorporation of ^19^F in more detail here.

Small angle scattering (SAS) and vibrational spectroscopies also rely on isotope enrichment to deduce specific information about regions of large molecules.^22^ SAS methods take advantage of solvent contrast variation of perdeuterated proteins by using buffers supplemented with specific D_2_O : H_2_O ratios.^23^ The resulting differences in scattering length densities between solvent and biomolecule allow the individual rendering of each (complex) part and its structural characterisation.^10^ For IR and Raman spectroscopies, replacement of specific bonded atoms with their isotopes allows assignment of the band arising from that specific bond in otherwise highly complex spectra. The assignment of the vibrational mode of a specific chemical group or amino acid residue enriched in ^13^C or ^15^N can generate valuable spatial dimensionality in the context of protein IR spectroscopy.^24^ Similarly, isotope enrichment enables the assignment of vibrational frequencies in Raman spectra to specific residues and therefore can contribute to the determination of the secondary structure or folding state of a protein.^25^

**Fig. 1:**
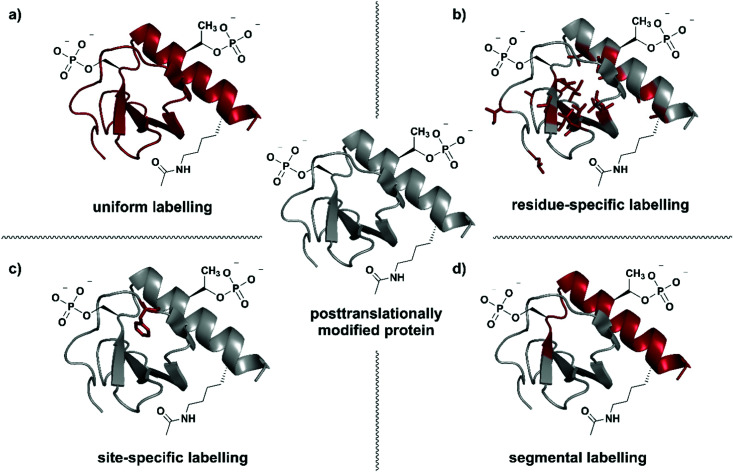
Isotope labelling strategies for posttranslationally modified proteins. (a) Uniform isotope labelling of a target protein in which either no or all protons, carbons and/or nitrogens are replaced by ^2^H, ^13^C or ^15^N (shown in red). (b) Residue-specific isotope labelling in which particular amino acid residues (*e.g.* Leu, Tyr, Met) are replaced by their isotope labelled counterparts, whereas the other residues remain unlabelled. (c) Site-specific labelling is the incorporation of one or more isotope labelled amino acid residues at certain key position(s) in an otherwise unlabelled polypeptide chain. (d) Segmental isotope labelling enables the incorporation of an isotope-labelled protein segment (peptide) into a larger protein. | The structure of Interleukin 8 (PDB: 3IL8) is used to illustrate the different labelling schemes. Protein parts with natural isotope abundance are shown in grey and isotope-enriched residues/segments in red.

In order to enrich an isotope in the protein of interest, several approaches based on providing the expression system with suitable sources of the required precursor molecules have been developed, yielding proteins that differ only in their isotopic composition (isotopologues).^26^ Typically, isotope labelling of proteins is achieved by recombinant production in a suitable expression system – typically *E. coli*, but also yeast, insect or mammalian cells.^26,27^ For the recombinant production of toxic or membrane proteins, cell-free expression can be employed.^28,29^ There are numerous experimental variations depending on the expression system and target protein, but typically minimal media for expression in bacteria are supplemented with ^13^C-glucose and/or ^15^NH_4_Cl as sole carbon and nitrogen sources for the incorporation of stable carbon and nitrogen isotopes.^26^ An alternative is Studier's glycerol-based autoinduction media.^30^ In uniform labelling schemes, nearly all atoms of a specific element in a protein (^12^C, ^14^N) are replaced with their respective isotopes (^13^C, ^15^N), typically leading to an isotope enrichment of more than 90% in the target protein (Fig. 1a).^26^ Deuteration (typically for larger proteins >25 kDa) can be achieved by replacing water with D_2_O and additionally by growing the expressing cells in media supplemented with ^2^H–^13^C-labelled glucose.^31,32^ However, deuteration is only applicable in prokaryotic cells (*E. coli* and *P. Pastoris*), as eukaryotes suffer from toxic isotope effects. As an alternative, one can use deuterated amino acid hydrolysates in combination with cell-free expression systems. Cell-free expression systems are particularly useful for amino-acid selective labelling,^33^ labelling of specific methyl groups,^34^ and stereo-array isotope labelling (SAIL).^35^ However, signal overlap in NMR spectra of large proteins can still prove challenging. Likewise, interpretation of IR and Raman spectra as well as SAS data is complicated by the number of signals arising from large proteins.^11,22,25^ In order to reduce spectral crowding and focus on particular regions of interest, it is sometimes necessary to apply more elaborate, tailored spectral simplification strategies, which will be discussed in more detail in the following sections.^17^

Selection of a specific isotope labelling strategy depends on the desired information, protein size and means of production. A reduction in the number of signals observed, for example in NMR spectra, can be achieved by either labelling specific groups (*e.g.* methyl groups), selected segments of a protein, or single amino acid residues at essential functional sites (Fig. 1). Such labelling results in more intense signals at the labelling site compared to their natural-abundance counterparts and therefore allows information on specific parts of the target protein to be obtained.

Residue-specific isotope labelling is achieved by supplying the expression host (*e.g. E. coli* grown on minimal media) with the respective labelled amino acid or its metabolic precursors (Fig. 1b). Labelled precursors for the desired amino acid are introduced into suitable amino acid synthesis pathways, resulting in labelling of all amino acid residues diverging from that point in the pathway.^26^ Methyl (–^13^C^1^H_3_) groups such as in valine, leucine, methionine, *etc.* are ideal candidates for the residue-specific incorporation of NMR-active isotopes.^36^ Methyl groups carry three protons and their symmetric arrangement yields intense and well-resolved NMR signals.^37^ Furthermore, the abundance of methyl functionalities in hydrophobic protein core regions and interaction sites makes them an attractive target for substitution with the respective isotopologues. Alternatively, Takeuchi *et al.* have demonstrated the usefulness of amino acids ^13^C-labelled at the carbonyl position against a ^2^H–^15^N background, which allows easy identification of these amino acids and shows much less scrambling compared to ^15^N labelling.^38^ Methyl-specific labelling takes advantage of the high sensitivity of the ^1^H nucleus at certain key sites in ^1^H–^13^C correlation spectra.^39^ The original ^13^C-labelling strategy for Ile, Leu and Val methyl groups was established by the Kay laboratory^40^ and developed further by the Boisbouvier group to allow differentiation between the respective methyl groups in NMR spectra by adding stereo-specifically labelled methyl groups.^41^ Other labelled biosynthetic precursors such as precursors for labelling the indole in Trp have been explored and are valuable tools for residue-specific isotope labelling.^42^ However, metabolic labelling processes used for residue specific labelling might cause potential scrambling of isotope labels due to metabolic interconversion of the amino acids.^43,44^

Labelling of single amino acid residues is a potential strategy when focusing on one or very few key positions in a protein. This strategy allows very simplified signal assignments and easy monitoring of reactions involving the labelled key residue(s), but comes with the disadvantage of ignoring the larger protein context (Fig. 1c). In this approach, isotope-labelled natural or unnatural amino acids are introduced site-specifically at the desired positions by protein (semi-)synthesis or genetic code expansion.^45,46^

Segmental isotope labelling is a strategy for reduction of spectral complexity that is applied to small and large proteins (Fig. 1d). This labelling strategy became possible with the introduction of expressed protein ligation (EPL) and protein *trans*-splicing (PTS), as well as by the use of enzyme-mediated ligation approaches.^47^ Labelled and unlabelled segments of the protein of interest are produced separately, with different uniform or residue-specific labelling patterns, and are subsequently chemically or chemoenzymatically ligated.^48–50^

## Protein semisynthesis by EPL

**Fig. 2:**
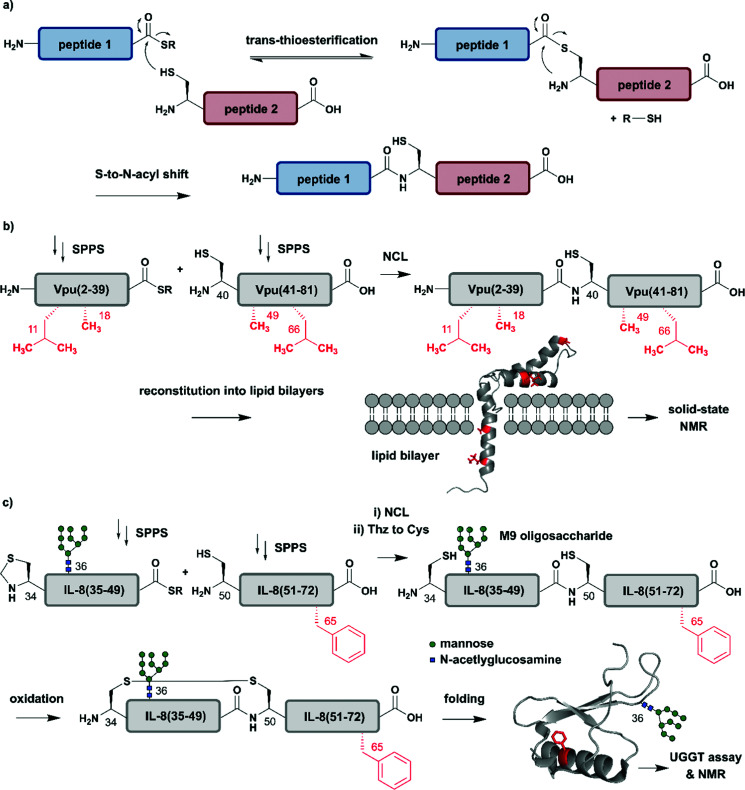
Chemical protein synthesis based on Native Chemical Ligation (NCL) for site-specific isotope labelling. All peptide segments were prepared by solid phase peptide synthesis (SPPS). (a) General mechanism of the NCL reaction. (b) Site-specific ^15^N-isotope labelling of four residues (red) of Vpu(2–81) (PDB: 2N28).^57^ (c) Synthesis of a IL-8(34–72) glycopeptide library varying Phe65 (red) with 10 different ^15^N-labelled amino acids by NCL. Site-specific isotope labelling with varying residues at the predicted core UDP-glucose:glycoprotein glucosyltransferase (UGGT) recognition site (Phe65) confirmed the crucial role of hydrophobic residues by NMR spectroscopy (PDB: 3IL8).^58^ | Grey: unlabelled protein parts | red: ^15^N isotope-labelled amino acid residues.

The incorporation of isotopologues into one or more protein segments, or at selected positions within a protein, requires connecting two (or more) separate polypeptides, ideally with a native amide bond so that the protein structure remains unaltered. Native Chemical Ligation (NCL, Fig. 2a) was developed in 1994 by Dawson *et al.* for linking unprotected peptide segments in aqueous conditions and has provided a route to access many modified proteins.^51^ Alternative amide-forming ligations include KAHA^52^ and Ser/Thr^53^ ligations; however, comparatively few proteins have been prepared using these strategies. As shown in Fig. 2a, an NCL reaction requires a C-terminal thioester functionality on one peptide segment. The other peptide segment bears an N-terminal cysteine residue. Upon ligation, a native amide bond is formed at the ligation site. The toolbox of extensions to NCL available today allows the rapid and efficient synthesis of proteins, and we direct readers to recent reviews on these topics.^47,54–56^

The high precision and scope of solid-phase peptide synthesis (SPPS) in combination with NCL enables the site-specific introduction of a broad range of modifications (including PTMs), non-natural amino acids, tags and isotope-enriched amino acids.^47^ In an early example, Kochendoerfer *et al.* demonstrated site-specific incorporation of isotope-enriched amino acids in 81-residue viral protein U (Vpu) by chemical synthesis and NCL (Fig. 2b).^57^ This integral membrane protein is crucial for virus particle release after human immunodeficiency virus-1 (HIV-1) infection through its modulation of viral protein trafficking and membrane permeability.^59^ Kochendoerfer *et al.* generated a ^15^N-labelled Vpu(2–81) analogue of this viral protein by ligating two site-specifically labelled peptide segments together. The first segment Vpu(2–39) bore ^15^N-enriched variants (isotopologues) of Leu11 and Ala18, and the second segment Vpu(40–81) contained these isotopologues at Ala49 and Leu66. After NCL and reconstitution into micelles and lipid bilayers, Kochendoerfer *et al.* assessed the overall topology and channel activity of their site-specifically labelled synthetic Vpu. ^1^H–^15^N HSQC (Heteronuclear Single Quantum Coherence) and solid-state NMR (ssNMR) experiments revealed comparable characteristics between the chemically synthesised version and the recombinantly expressed, uniformly labelled control protein (without site-specific labelling). ^15^N-labelling of the protein backbone at four specific residue sites allowed very simplified signal assignments compared to the uniformly labelled Vpu variant. The site-specific labelling pattern provided a rapid fingerprint of membrane-embedded Vpu in ssNMR measurements, in which only the four ^15^N-labelled residues could be detected.^57^ Although lacking PTMs, Vpu is an example of how site-specific incorporation of NMR-active nuclei can be used to report on key positions in protein structure and function, especially in membrane systems studied by ssNMR.

Incorporation of both isotope enriched building blocks and posttranslationally modified residues was demonstrated for glycosylated interleukin 8 (IL-8) (Fig. 2c).^58^ In general, glycosylation is a PTM involving covalent attachment of specific oligosaccharide units varying in length and composition to amino acid sidechains by N- and O-linkages, in the endoplasmic reticulum (ER) and Golgi.^60^ Glycans themselves can also be characterised by NMR spectroscopy, even without specific labelling or elaborate sample preparation.^61^ The example selected here by Izumi *et al.* focuses on the enzyme UDP-glucose:glycoprotein glucosyltransferase (UGGT) that acts as a quality-control system for protein folding in the ER. To study the mechanism by which UGGT recognizes misfolded species of N-glycosylated IL-8, Izumi *et al.* constructed an IL-8 glycopeptide library using NCL.^58^ IL-8 was split into two segments, with the N-terminal segment IL-8(34–49) bearing a Man9GlcNAc2 (M9) oligosaccharide at Asn36 and the C-terminal segment IL-8(50–72). To identify possible hydrophobic patches recognized by UGGT, Izumi *et al.* selected specific residues and incorporated ^15^N-labelled variants at the respective positions. After NCL and subsequent air oxidation, the resulting folded IL-8(34–72) was analysed by ^1^H–^15^N HSQC NMR and revealed a significant chemical shift perturbation of Phe65 upon binding to UGGT. To further investigate the hydrophobic interaction patch at this position, the authors generated a glycopeptide library by substituting Phe65 with ten labelled natural amino acids (Glu, Lys, Gln, Ser, Tyr, Gly, Ala, Pro, Leu and Val). Based on this library generated using NCL, the authors concluded that glycopeptide variants bearing residues with greater hydrophobicity are glycosylated and folded faster.^58^ These chemically synthesised glycopeptides bearing a ^15^N label at specific positions are an excellent example of combining site-specific isotope-labelling with installation of complex PTMs. As only selected residues are labelled, and therefore observed, assignment of signals in NMR spectra can be simpler than assigning signals from all residues. The obvious drawback is that interactions or conformational changes involving unlabelled residues cannot be assessed easily. Furthermore, the total chemical synthesis of proteins for spectroscopic applications suffers from restrictions in length of the accessible proteins and prohibitively expensive isotope-labelled amino acid building blocks.^62^ Therefore, SPPS is more widely used to incorporate site-specific PTMs than site-specific isotope labels.

Incorporation of site-specific isotope labels can also be achieved by the use of auxotrophic bacterial strains, which require the specific supplement of the respective labelled amino acid in their growth media. Although this methodology can be applied to almost all of the spectroscopic techniques described in this review, it is predominantly used for studying complex systems such as membrane proteins by IR spectroscopy.^63^ To this end, bacteriorhodopsin was generated with all 7 Arg residues labelled with ^15^N in an auxotrophic *Halobacterium salinarium* strain.^64^ Comparison of the vibrational differences in IR spectra between unlabelled and ^15^N-labelled samples delivered valuable insights and helped to identify Arg82 as a key residue in the light-induced H^+^ transport mechanism. Although residue-specific labelling *via* auxotrophic bacteria strains can be performed with relative ease, the approach lacks the precision of protein semisynthesis or genetic code expansion and therefore is only applied occasionally.

**Fig. 3:**
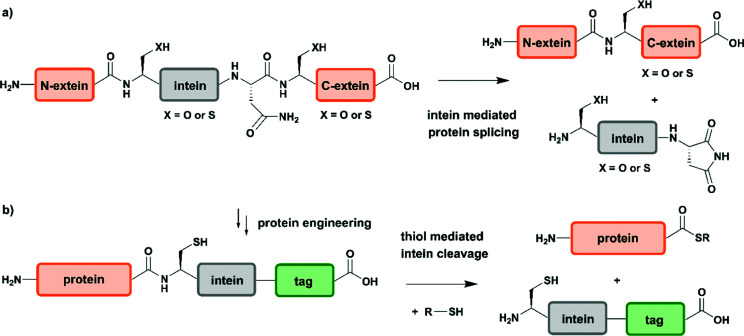
Intein-mediated protein splicing and generation of protein thioesters. (a) General mechanism of protein splicing. Intein-mediated protein splicing results in linkage of the N- and C-extein (orange) by a native peptide bond after excision of the intein functionality. (b) Protein engineering can generate extein–intein fusion proteins bearing C-terminal purification tags (green), which yields protein α-thioesters after addition of a thiol. | Grey: intein | orange: N-extein and C-extein | green: affinity purification tag.

Two years after introducing NCL for linking unprotected synthetic peptide segments by a native amide bond, the reaction was extended to the N-terminal modification of a recombinantly expressed protein carrying an N-terminal Cys residue with a thioester-containing fluorescent dye.^65^ In 1998 NCL was used for ligation of recombinantly produced α-thioesters to synthetic peptides, a process named expressed protein ligation (EPL).^66,67^ Recombinant protein expression enables access to protein segments without size limitations and various isotope patterns can be incorporated using methods discussed above. EPL can therefore be used to ligate a recombinantly expressed (and isotope-labelled) protein segment to a synthetic segment bearing a site-specific PTM.^68^ Several methods to generate recombinant protein segments with N-terminal Cys residues have been used in protein semisynthesis. Direct access to such segments is possible if cleavage of the expressed protein segment by endogenous methionine aminopeptidase or signal peptidases occurs. Other strategies rely on selective cleavage of Met–Cys with cyanogen bromide or the use of proteases with highly defined recognition sequences such as factor Xa, thrombin, tobacco etch virus (TEV) protease and small ubiquitin-like modifier (SUMO) protease.^56^ Access to recombinant protein segments with C-terminal α-thioesters makes use of protein splicing.^66^ The underlying biochemical reaction is based on an autocatalytic process controlled by inteins (internal or intervening protein) that induce protein splicing events in which peptide bonds are cleaved and formed (Fig. 3a)^69,70^ and results in splicing of the intein from its N- and C-terminal flanking polypeptides (exteins – external proteins) that are linked by an amide bond – also termed protein splicing (in *cis*).^56^

Since the discovery of inteins and their splicing mechanisms, protein engineering attempts have exploited the versatility of inteins for protein purification to generate proteins with N-terminal cysteine residues and for accessing proteins with C-terminal α-thioesters that can be used in NCL reactions (Fig. 3b). Tags such as the chitin-binding domain and/or poly-histidine tags allow for affinity purification of proteins followed by thiol-mediated intein cleavage, ideally resulting in pure protein segments carrying C-terminal α-thioesters.^71^ Among the large number of inteins found to date in all kingdoms of life, the DNA gyrase A (*Mxe* GyrA) intein from *Mycobacterium xenopi* has been most frequently employed for the generation of protein α-thioesters.^72^ However, inteins that show faster cleavage kinetics and higher tolerance towards varying flanking sequences have been reported.^73,74^ Combining large recombinant protein segments with tailor-made synthetic peptides has been successfully exploited for segmental labelling of proteins carrying common PTMs such as phosphorylation, acetylation or ubiquitination.

**Fig. 4:**
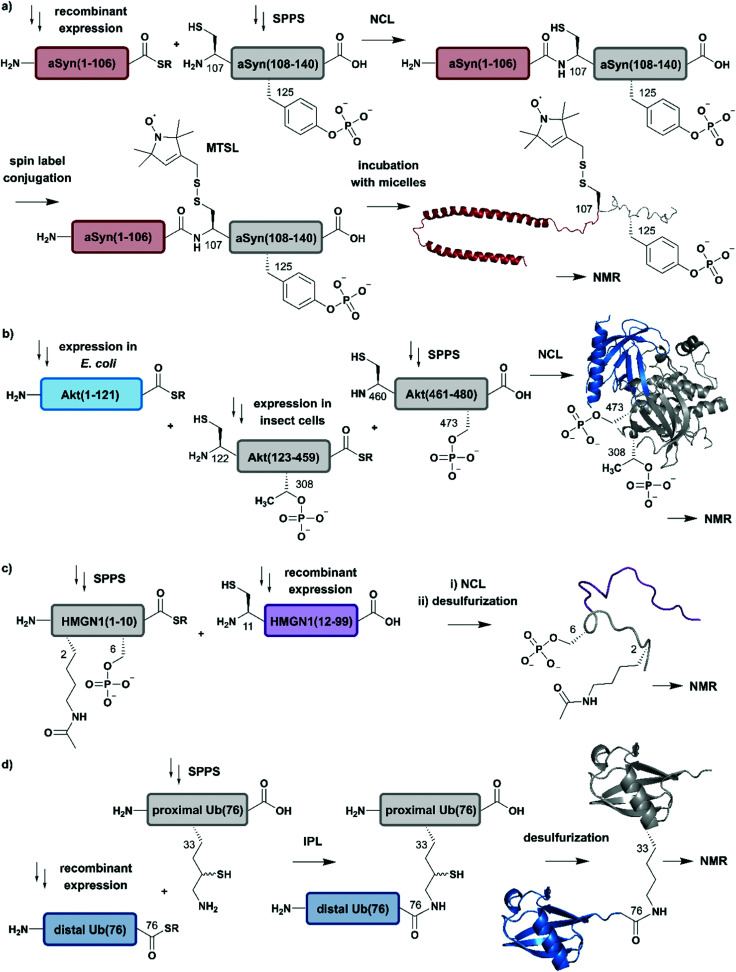
Expressed Protein Ligation (EPL) for segmental isotope labelling of proteins. (a) Segmental isotope labelling of posttranslationally-modified αSyn(1–140). Recombinant expression of αSyn(1–106) (red) allowed homogenous incorporation of isotopes into the N-terminal αSyn(1–106) segment and chemical synthesis gave access to the C-terminal peptide containing phosphorylated Tyr125. Upon ligation phosphorylated and segmentally labelled αSyn enabled the detailed study of the influence of Tyr125 phosphorylation on the protein by NMR spectroscopy (PDB: 1XQ8).^75^ (b) Segmental labelling of posttranslationally modified Akt(1–480) by protein semisynthesis. The three-segment assembly strategy is worth mentioning as the two recombinantly produced segments are generated in two different expression systems (bacteria and insect cells). Isotope labelling of Akt(1–121) (light blue) was achieved in *E. coli* (PDB: 3O96).^83^ (c) Segmental labelling and site-specific modification of HMGN1(1–99) by protein semisynthesis. SPPS allowed the site-specific introduction of selected PTMs (acetylation and phosphorylation) in HMGN1(1–10) (grey) and recombinant isotope-labelled HMGN1(11–99) (purple) segment revealed long-range chemical shift perturbation in subsequent NMR studies (PDB of HMGB: 1LWM).^84^ (d) Isopeptide chemical ligation (ICL) approach for the isotope labelling of the distal Ub(1–76) subunit (blue) in a dimeric Ub chain. The synthetically-derived proximal Ub (grey) was equipped with δ-mercaptolysine at Lys33 and ligated to the recombinant distal Ub subunit, which enabled Castañeda *et al.* to describe the characteristics of a distal Ub unit in a dimeric Ub assembly using NMR spectroscopy (PDB: 1UBQ).^85^ | Grey: unlabelled protein parts | red/light blue/purple/blue: homogenous, stable isotope-labelled protein segments.

Advantage was taken of EPL in the semisynthetic approach from Hejjaoui *et al.* towards α-synuclein (αSyn), in which the authors sought to introduce phosphorylation(s) site-selectively with segmental isotope labeling of the protein. More precisely, the authors investigated the site-specific phosphorylation of αSyn at Tyr125 (Fig. 4a).^75^ Phosphorylation can affect protein structure and function, as has been shown for many cellular processes and also in several semisynthetic approaches for αSyn,^75,76^ which plays a central role in Parkinson's disease.^77^ Phosphorylation usually occurs on sidechain hydroxyl groups of Ser, Thr and Tyr. Removal of the phosphate group is mediated by the smaller family of protein phosphatases.^78^ In NMR spectra, protein phosphorylation results in a significant downfield shift of Ser (and/or Thr/Tyr) backbone amide resonances.^79,80^ In the study from Hejjaoui *et al.*, a synthetic, phosphorylated αSyn segment (residues 107–140) was ligated to its recombinantly expressed N-terminal counterpart. The recombinant segment was expressed as a αSyn(1–106)–*Mxe* GyrA intein–6xHis fusion protein in M9 minimal medium, supplemented with the sole nitrogen source ^15^NH_4_Cl. The C-terminal α-thioester functionality was generated through MESNa (2-mercaptoethanosulfonate)-mediated intein splicing directly after Ni–NTA purification. The C-terminal αSyn segment, comprising residues 107 to 140 (containing an Ala107Cys mutation to allow EPL) and bearing either no or a single phosphorylation at Tyr125, was produced *via* SPPS. The resulting semi-synthetic, segmentally isotope labelled wildtype (wt) and phosphorylated Tyr125 (pTyr125) αSyn(1–140) variants were conjugated with a methanethiosulfonate (MTSL) spin label at the Cys residue 107 (resulting from the NCL reaction) for NMR-based paramagnetic relaxation (PRE) experiments.^81,82^ The authors characterised both MTSL-conjugated αSyn variants in their micelle-bound state by NMR and compared biological aspects such as fibrillization. The data revealed significant differences between variants in their monomeric state and suggested a lower local compactness of pTyr125 αSyn compared to the unmodified aSyn. The semisynthetic strategy allowed Hejjaoui *et al.*, to investigate the influence of pTyr125 on αSyn structure, aggregation, membrane binding and subcellular localisation.^75^ This work is an excellent example of the power of protein semisynthesis in studying proteins bearing homogeneous, site-specific PTMs and their impact on transient long-range interactions. The NMR-active ^15^N-labelled αSyn(1–106) segment provides insight into the effects of pTyr125 on the αSyn structure in solution and in membrane proximity at atomic resolution. The synthetic, unlabelled C-terminal segment is not visible in ^1^H–^15^N NMR spectra and therefore simplifies assignment of the signals arising from the recombinant (labelled) segment.

In another study, which combined protein semisynthesis, segmental isotope labelling and enzymatic phosphorylation, Chu *et al.* sought to characterise the molecular determinants of the (in-)activation of the AKT serine/threonine kinase 1 (AKT) at atomic resolution (Fig. 4b).^83^ Activation of the AKT protein (480 residues) is modulated by phosphorylation events at its C-terminal domain, which lead to a relieved intramolecular interaction of its N-terminal pleckstrin homology (PH) domain from the central kinase domain.^86^ To decipher the key determinants of this interaction, the authors applied a two-step NCL strategy for the generation of the three domain Akt protein kinase, which required mutation of Ser122 to Cys to enable segmental labelling *via* a chemoselective ligation reaction. The SPPS-derived C-terminal segment Akt(460–480) allowed the generation of the respective phospho-forms, yielding peptides bearing phosphate groups at pSer473 or pSer477/pThr479. Unlabelled, non-deuterated Akt(122–459) kinase domain was obtained recombinantly from *Sf9* insect cells, which were phosphorylated *in vitro* by GST-PDK1 at pThr308 and then ligated to the C-terminal Akt(460–480) segment. Isotope labelling of the N-terminal PH domain Akt(1–121) with ^2^H, ^13^C and ^15^N isotopes was achieved by expression in M9 minimal media using an *E. coli* expression system, with ^2^H–^13^C glucose and ^15^NH_4_Cl as sole carbon and nitrogen sources in D_2_O-supplemented media. In a second NCL reaction, labelled Akt(1–121) was ligated to the previously generated Akt(122–480) and characterised by ^1^H–^15^N HSQC NMR. By combining chemical shift perturbations with peak broadening in the resulting NMR spectra, the data revealed strong interaction patterns between the PH domain and the unlabelled Akt(122–480) segment. Segmental labelling of Akt(1–121) greatly reduced spectral crowding in these experiments and therefore allowed resonance assignment of 66% of non-proline backbone residues in the PH domain, enabling the detailed study of long-range intramolecular interactions between these Akt domains at atomic resolution. These interactions were highly dependent on the respective phosphorylation state of the C-terminal Akt(460–480) segment, which were additionally characterised by cross saturation transfer (CST) NMR measurements.^87^ This CST experiment monitors the selective transfer of magnetization from the protonated Akt(123–459) kinase domain to the perdeuterated N-terminal Akt(1–122). Using this measurement, the authors were able to distinguish between direct (transfer of magnetization) and indirect (no transfer of magnetization) interaction patterns within those two Akt domains. The CST data revealed an influence of pSer473 pSer477/pThr479 on Akt kinase activity and additionally highlighted the importance of a particular linker region spacing the PH domain from the kinase domain, which was already identified in a previous study from the same group^88^ and characterised in detail.^83^

In a further example of segmental labelling by protein semisynthesis, the effects of site-specific acetylation and phosphorylation on the nucleosome-binding protein HMGN1 were recently investigated (Fig. 4c).^84^ Lys acetylation is catalysed by lysine acetyltransferases (KATs) and reversed by lysine deacetylases (KDACs).^89^ Both enzymatic reactions can be monitored by NMR in real time, as they exhibit characteristic features on the Lys Nα and Nε in ^1^H–^15^N correlation experiments.^90^ Niederacher *et al.* used EPL for the generation of several segmentally isotope-labelled HMGN1 variants bearing site-specific PTMs within the N- and C-terminal parts of the protein. N-terminal acetylation at Lys2 (acLys2) and phosphorylation at Ser6 (pSer6) were introduced in a peptide comprising residues 1–10 of HMGN1(1–10).^84^ After ligation of the N-terminal segment to the isotope-labelled, recombinant HMGN1(11–99) segment, the product was characterised by NMR spectroscopy. ^1^H–^15^N HSQC NMR experiments revealed long-range perturbations in the chemical environment of the backbone residues, even in this intrinsically disordered protein (IDP). Furthermore, segmental labelling here helped to minimize signal overlap in the “random coil” region of the ^1^H–^15^N correlation spectra, which is often crowded for uniformly labelled IDPs.^91^ In particular, the variant bearing both modifications showed significant shifts in the nucleosome-binding domain, which suggests an influence on the binding affinity of HMGN1 to nucleosomes even for small PTMs like acetylation or phosphorylation. This study is an example of how protein semisynthesis can flexibly provide a set of isotope-labelled and PTM-carrying protein variants (modified at their N- or C-termini) that offer valuable insights into the effects of selected PTMs on protein structure and function at the atomic level. The segmental labelling approach facilitated resonance assignment and led to insights into PTM-modulated structural changes and functions.^84^

Another frequent PTM characterised using a combination of ligation chemistry and spectroscopic investigations is ubiquitination. Ubiquitination is the covalent attachment of ubiquitin (Ub) or polyubiquitin (polyUb) chains to the Lys ε-amino group of a target protein. Several synthesis strategies have been used for the generation of (poly-)ubiquitin and its installation on target proteins,^92^ but we focus here on Ub attachment combined with NMR spectroscopic analysis. The repetitive nature of polyUb molecules makes it almost impossible to resolve the respective NMR signals from individual subunits, which remained a challenge until 2011.^93^ This drawback was addressed first by Castañeda *et al.* by applying an isopeptide chemical ligation approach in order to generate segmentally isotope-labelled, dimeric Ub (diUb) (Fig. 4d).^85^ In this study, the proximal Ub was chemically synthesised with a δ-mercaptolysine at Lys33.^94^ The incorporation of NMR-active isotopes (^15^N) in the distal Ub molecules was achieved by recombinant expression in auto-inducing minimal media. After a successful ligation reaction and the following rearrangement *via* S-to-N acyl shift, the native diUb was desulfurised and characterised by NMR spectroscopy. This allowed Castañeda *et al.* to completely remove signal overlap from the individual diUb elements and revealed features about the structure, conformation and ligand-binding of Lys33-linked diUb. The experiments provided insight onto the monomer-specific inter- and intramolecular interactions of the respective Ub subunits.^85^ Although this approach involves isotope-labelling of the PTM (ubiquitin) rather than the substrate protein, it illustrates the power of protein semisynthesis for studying PTMs by NMR. Without the combination of peptide synthesis, which allowed the site-specific incorporation of the required δ-mercaptolysine, with isotope labelling by recombinant expression, such a detailed characterisation would have not been possible. Furthermore, the principle of segmental labelling of individual Ub subunits paved the way for non-natively linked Lys11-, Lys27- or Lys63 polyUb chains, as discussed later in this review.

In summary, the currently-available toolbox for generating N-terminal cysteinyl and C-terminal α-thioester peptide/protein segments *via* chemical synthesis and recombinant expression allows the detailed study of posttranslationally modified proteins by NMR spectroscopy. Although other spectroscopic techniques such as Raman or IR spectroscopy greatly benefit from segmental labelling including deuteration by EPL, no relevant examples in the context of PTMs have been published so far. However, we direct the reader to examples of segmental labelling for other spectroscopic techniques,^95–97^ which suggest potential future applications.

## Protein *trans*-splicing (PTS)

**Fig. 5:**
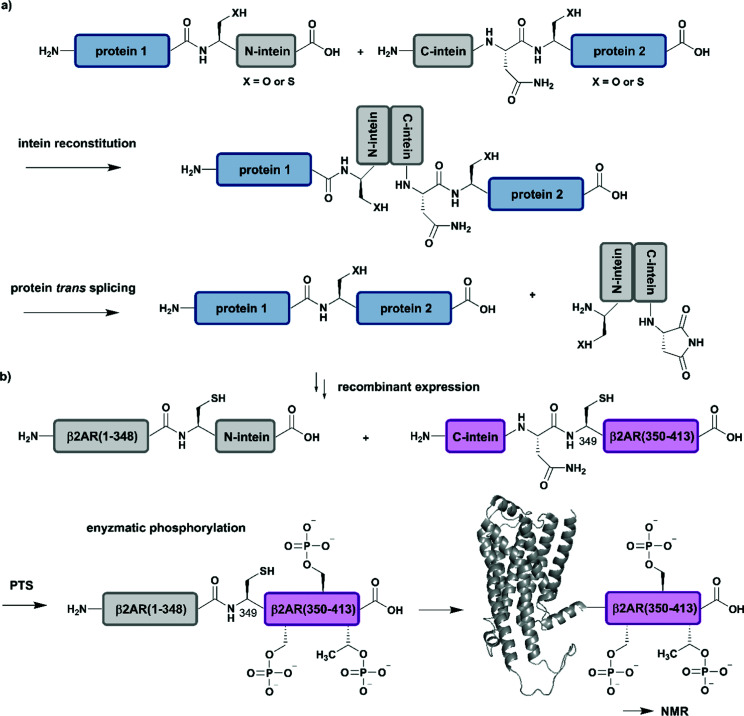
Protein *trans*-splicing (PTS) for segmental isotope labelling. All protein segments were prepared by recombinant expression. (a) General mechanism for the PTS reaction. (b) Segmental isotope labelling of enzymatically phosphorylated β2AR(1–413) by PTS. The unlabelled β2AR(1–348)–N-intein fusion (grey) construct and its isotope labelled counterpart C-intein-β2AR(349–413) (purple) were recombinantly expressed, ligated by PTS, enzymatically phosphorylated and investigated by NMR spectroscopy (PDB: 6KR8).^103^ | Grey: unlabelled protein parts | purple: homogenous, stable isotope-labelled protein segments.

Intein-mediated protein splicing in *cis* relies on a continuous protein segment flanked by the N-extein and C-extein sequences, however there are also discontinuous intein variants (so-called split inteins), which can be exploited for segmental labelling of proteins by semisynthesis.^98^ These split inteins are encoded on different gene loci and associate with high affinities in the nanomolar range. Upon association of the split intein segments, a splicing reaction of the intein through a “capture and collapse” mechanism is initiated, named protein *trans*-splicing (PTS) (Fig. 5a).^99^ Natural split inteins are somewhat restricted in use due to their strict sequence requirements. Extensive split intein engineering has led to several shorter intein sequences, novel PTS triggers, faster splicing reactions and also increasing amino acid tolerance in the terminal parts of the exteins.^100^ Whereas a broad range of C-terminal split intein domains with an appropriate sequence length (∼36–50 amino acid residues) for SPPS are naturally available and can be used for the incorporation of PTMs and other modifications including specific isotope labels, recent engineering attempts have focused on the reduction of the sequence length of the N-terminal intein domain.^101^ Whereas the sequence requirements for PTS are typically more restrictive than for EPL methodology, PTS can be carried out at significantly lower concentrations due to the high affinities of the split intein segments.^47^ Furthermore, split inteins can also be made dependent on various external stimuli and exploited for conditional protein splicing *in vitro* as well as *in vivo* reactions with dimerizing molecules, pH or light.^102^

Application of PTS to the study of structural and functional effects of phosphorylation is demonstrated by a study on the G-protein-coupled receptor (GPCR) β2AR (β2-ardrenoreceptor). The binding of agonists to the extracellular domain of GPCRs, which constitute the largest group of cell surface receptors, leads to phosphorylation of their C-terminal cytoplasmic region. Shiraishi *et al.* investigated conformational changes and altered membrane interactions due to phosphorylation of the β2-ardrenoreceptor (β2AR) (Fig. 5b).^103^ The authors created a β2AR variant bearing a segmentally labelled C-terminal tail, which was subsequently studied by NMR spectroscopy. Shiraishi *et al.* achieved this by using an intein-mediated PTS approach, which allowed them to conjugate unlabelled β2AR(1–348) and ^2^H–^13^C–^15^N-labelled β2AR(349–413) with ∼80% efficiency. Both fusion constructs bearing DnaE split inteins, β2AR(1–348)–N-intein as well as C-intein-β2AR(349–413), originated from recombinant expression systems and provide the possibility for isotope labelling using amino acids or metabolic precursors. To suppress signal overlap, they selectively labelled C-terminal Thr and Ile methyl groups using growth media with [α,β-^2^H_2_, γ2-^13^C^1^H_3_] Thr, [^2^H_2_] Gly and [methyl-^13^C, 3,3-^2^H] ketobutytric acid as appropriate precursor molecules. The resulting segmentally labelled, full-length β2AR(1–413) was embedded into lipid nanodiscs and phosphorylated by GPCR kinase 2. This is one of the few cases in which a specific kinase is available to introduce multiple phosphorylations site-specifically into a large protein. NMR studies revealed a phosphorylation-dependent interaction of the C-terminal tail of β2AR with intracellular membrane compartments. Following on from a previous study on labelling of Met residues,^104^ Shiraishi *et al.* further investigated their findings by specific labelling of four Met residues in a 4Met β2AR mutant with [^2^H-9AA, αβγ-^2^H-, methyl-^13^C-Met] precursor molecules. ^13^C-labelling of methionine methyl groups is a commonly used methodology in protein NMR and allowed them to assess the structural conformation of the transmembrane helices of the phosphorylated species. Shiraishi *et al.* proposed that phosphorylation might result in an allosteric rearrangement of the transmembrane helices and therefore lead to formation of the structural motif for β-arrestin binding, which offers a possible conserved structural pattern for GPCR recognition.^103^ Here, the authors impressively demonstrate how to overcome the challenges of generating and folding an integral membrane protein after PTS and how to combine PTS with a specific kinase and reconstitution into lipid nanodiscs to collect NMR data that further improves our understanding of signaling events at the molecular level.

To summarize, the discovery, engineering and application of intein-mediated PTS has led to an extensive repertoire of methods for protein assembly. The PTS toolbox allows for the design, synthesis/expression and assembly of synthetically-derived peptides and recombinantly expressed proteins at much lower concentrations than are typically possible in NCL, for example. This allows for segmental isotope labelling and site-specific incorporation of PTMs, fluorescents tags or other modifications for structural studies including those on challenging protein targets such as membrane proteins. PTS can easily be extended to *in vivo* cell experiments, as the PTS reaction is highly biorthogonal.^105,106^ However, PTS systems are still dependent on the flanking sequence of the C-extein and suffer from cross-labelling in *in vivo* segmental isotope labelling approaches,^107^ which restricts their application. Possibilities to circumvent these drawbacks are the use of orthogonal methods such as chemoselective ligations other than NCL or the use of specific enzymes such as Sortase A, which we will discuss below.

## Enzyme-mediated protein ligation

**Fig. 6:**
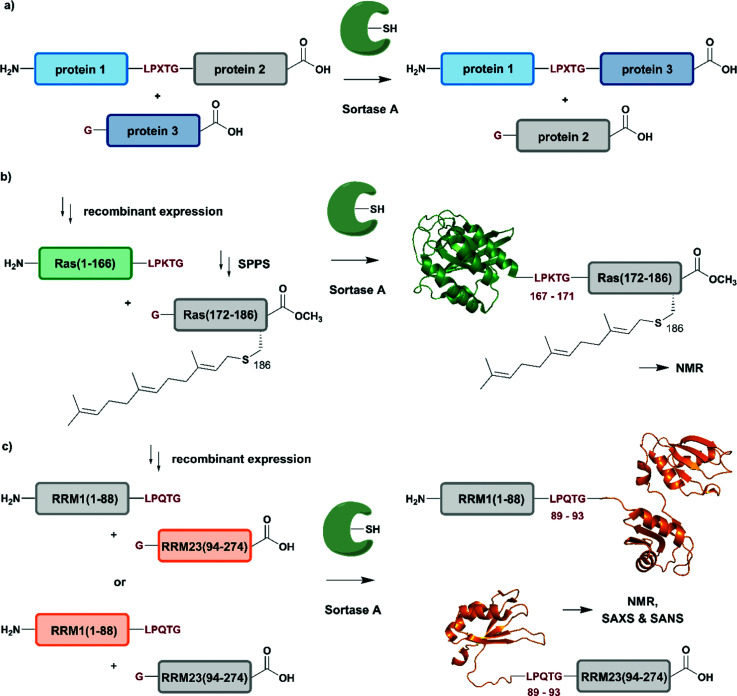
Sortase A-mediated protein ligation (“sortagging”) for segmental isotope labelling. (a) The transpeptidase Sortase A recognizes an “LPXTG” motif (in red) and catalyses an acyl transfer reaction *via* an acyl-enzyme intermediate. (b) Segmental isotope labelling of farnesylated Ras(1–186) (green) by employing Sortase A. Stable isotope labelled Ras(1–166) was ligated to the C-terminal Ras(172–186) (grey) peptide and subsequently characterised by NMR (PDB: 5X9S).^117^ (c) Segmental deuteration and isotope labelling of RRM(1–274) by sortagging for NMR, SAXS and SANS experiments. Sortase A allowed the incorporation of stable isotopes either in the N-terminal RRM(1–88) or the C-terminal RRM23(94–274) domains. (PDB: 5O2V for RRM1; 5O3J for RRM2; 1X4G for RRM3).^118^ | Olive: schematic Sortase A enzyme with its catalytic Cys residue | grey: unlabelled protein parts | green/orange: homogenous, stable isotope-labelled protein segments | red: amino acid substitutions at the ligation junction.

Naturally occurring and engineered enzymes that ligate two protein segments together provide an alternative methodology for ligating protein segments bearing PTMs and/or isotope labels. One of the most widely used, the transpeptidase Sortase A from *Staphylococcus aureus*,^108^ has been exploited for cyclization, bioconjugation and ligation of polypeptides.^109^ In the transpeptidation mechanism of this enzyme, the thiol nucleophile of a catalytic Cys residue attacks the Thr–Gly peptide bond in a substrate containing the *P*_1_-LPXTG-*P*_2_ motif (*P*_1_/*P*_2_/*P*_3_ = residues flanking the ligation site; X = any amino acid) (Fig. 6a and Table 1). The resulting acyl–thioester–enzyme complex is attacked by a nucleophilic polypeptide (G-*P*_3_) carrying one or more N-terminal Gly residues. This reaction, also referred to as ‘sortagging’, results in the formation of the *P*_1_-LPXTG-*P*_3_ ligation product, which bears a native peptide bond at the ligation site.^109^ Sortagging is an efficient tool for bioconjugation and has been used for segmental isotope labelling of multi-domain proteins such as vinculin^110^ or the attachment of solubility tags.^111^ However, the ligated products bear the LPXTG motif as a ligation “scar” if this is not part of the native sequence at the ligation site. Further drawbacks are the poor catalytic efficiency of the enzyme and reversibility of the transpeptidation reaction as it generates a Sortase recognition site upon ligation, which can reduce the reaction yield.^112^ Therefore, Sortase A homologs and engineered variants have been investigated to overcome these sortagging scars and the limited catalytic efficiency.^113,114^ Overall, Sortase A-catalyzed reactions can provide milligram quantities of ligated product and allow the introduction of PTMs, which makes them an attractive tool for chemoenzymatic segmental labelling of proteins.^115,116^

**Table 1:** Peptidyl transferases and peptide ligases

Enyzme and recognition site	Advantages	Disadvantages
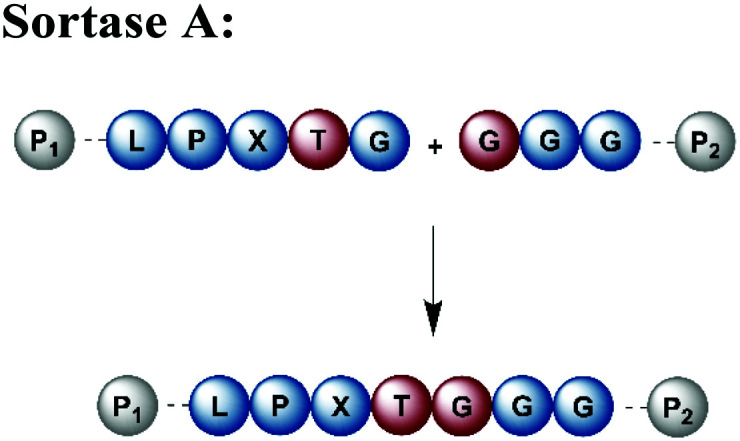	• Robust to a broad range of conditions	• Not traceless (LPXTG scar)
	• High yields possible	• Low catalytic efficiency
	• Recombinant expression possible	• Reversible reaction and hydrolysis
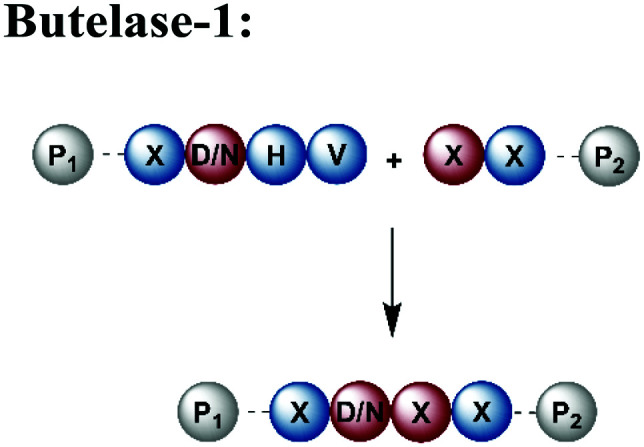	• Smaller recognition site than Sortase-A	• Not traceless (D/N scar)
	• High yields and efficiency	• Recombinant expression not possible
	• Broad sequence acyl acceptor	• Reversible reaction and hydrolysis
	• Recombinant expression possible (only for OaAEP1b)	• Intermediate yields and poor efficiency (only for OaAEP1b)
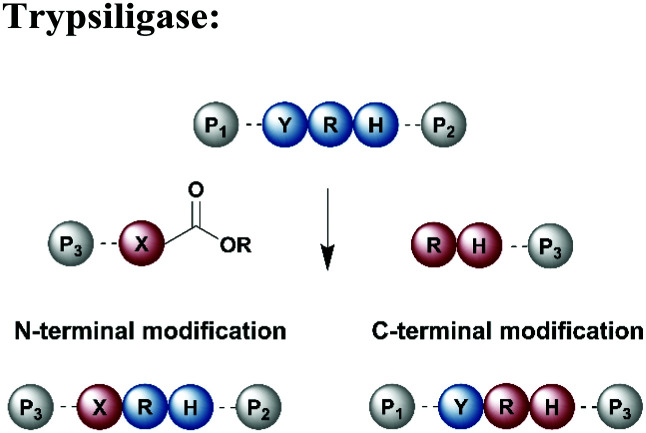	• Fast reaction (few minutes)	• Not traceless (Y-RH scar)
	• High substrate selectivity	• Strict sequence dependency
	• N- and C-terminal modifications possible	• Reversible reaction and hydrolysis
	• Recombinant expression possible	• Activated ester required
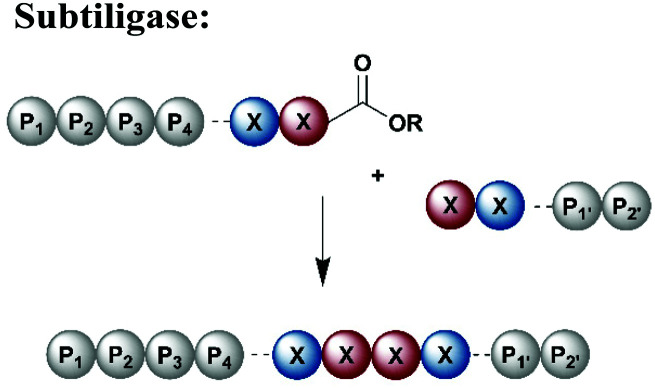	• Broad sequence compatibility	• Reversible reaction with some variants
	• Reduced hydrolysis compared to other enzymes	• Activated ester required
	• High catalytic efficiency	
	• Recombinant expression possible	

The advantages of segmental labelling of proteins by sortagging were demonstrated in a study by Ke *et al.*, where the strategy was combined with lipidation of the rat sarcoma (Ras) GTPase (Fig. 6b).^117^ Lipidations play central roles in controlling protein localisation, in protein secretion, signal transduction or apoptosis and are therefore an important, but also challenging target (due to handling problems) for structural investigations.^119,120^ Prenylation and palmitoylation are found on a broad range of target proteins, with the rat sarcoma (Ras) GTPase family being a prominent substrate for these modifications.^121^ Lipidation of the Ras protein mainly occurs in its C-terminal hypervariable region Ras(172–186) and triggers several modification processes, which result in a farnesylated Ras(1–186) variant bearing a C-terminal carboxymethylation. This process leads to localisation of Ras at the cell membrane, a prerequisite for subsequent activation of downstream kinases such as c-Raf-1.^122^ To decipher the molecular key determinants of this interaction by NMR, beyond the analysis based on fluorescence developed earlier,^123,124^ Ke *et al.* utilized a versatile Sortase-A based ligation strategy for the preparation of C-terminally modified Ras (Fig. 6b).^117^ The authors recombinantly expressed the catalytic domain of Ras(1–166) in M9 minimal medium, which allowed them to label this segment with ^13^C and ^15^N. The Ras(1–166) fusion protein was N-terminally conjugated to a 6xHis-tag for affinity purification and bore the required LPKTG motif for sortagging at its C-terminus. The synthetic Ras(172–186) peptide variants carried a farnesylation at Cys186 and an N-terminal Gly nucleophile, which is required for the Sortase-A mediated ligation. The ligation reaction was performed with an excess of Sortase A and the peptide-terminal segment to accommodate for the limited catalytic efficiency of the enzyme and to address further drawbacks such as product hydrolysis and undesired reverse reactions. Furthermore, the reaction required mutation of residues 167–171 in the Ras sequence to the non-native LPKTG sequence, which might alter the flexibility of the protein despite its non-native origin.^125^ With the prenylated Ras variants carrying different PTM patterns in hand, Ke *et al.* characterised the intramolecular interaction between Ras(172–186) and its catalytic domains by NMR. ^1^H–^15^N HSQC experiments revealed several signals with significant chemical shift perturbations and led to the identification of intramolecular interaction sites between the catalytic Ras(1–166) domain and the C-terminal Ras(172–186) segment. Further experiments combined NMR spectroscopy with surface plasmon resonance and an increased binding affinity was observed between the catalytic Ras domain and the C-terminal Ras(172–186) segment upon farnesylation,^117^ in accordance with previously published results from others.^126^ This work showcases an application of Sortase A-mediated protein ligations with challenging peptide substrates. The relatively small modification to the Ras sequence (compared to fusion constructs with inteins in EPL and PTS approaches) is straightforward and typically does not require extensive optimization, making it quite popular within the NMR community for segmental labeling approaches.

Segmental labelling of proteins also plays an important role when studying the shape of macromolecular assemblies using SAS technologies. By combining perdeuteration of certain segments with contrast variation with the solvent, the position of each individual protein subunit in such assemblies can be revealed, as demonstrated by Sonntag *et al.* (Fig. 6c).^118^ The authors applied a segmental, domain-selective perdeuteration approach for a structural study of the RNA-binding protein T-cell-restricted intracellular antigen-1 (TIA-1) using Sortase A-mediated ligations. TIA-1 comprises three RNA recognition motifs (RRM), which were split into two RRM segments (RRM1(1–92) and RRM23(93–274)).^127^ The segments are connected *via* a flexible linker region, which was chosen as a ligation junction for sortagging. In addition to an N-terminal 6xHis-tag and TEV protease cleavage site, RRM1(1–92) carried the LPQTG motif required for sortagging at its C-terminus. RRM23(93–274) bore a Gly residue at its N-terminus. Due to the possible drawbacks of this sequence manipulation, the authors carefully analysed the properties of native and ligated RRM123 upon RNA binding by comparing both variants using ^1^H–^15^N HSQC NMR spectroscopy, SAXS and static light scattering. As no differences were observed between the variants, the sortagging protocol was applied to segmental deuteration of either RRM1(1–92) or RRM23(93–274) (Fig. 6c). The initial structural analysis and computational modelling of both segmentally deuterated RRM123 versions revealed approximately 5000 different structural models of the protein–RNA-complex. After the use of SAXS and contrast-matched SANS filters following the initial NMR experiments, the structure ensemble could be refined to only 5 models of the RRM123–RNA complex.^118^ Although this study did not include any PTM, it represents an important example of how segmental isotope labelling of proteins can provide insight into individual subunits of multi-domain proteins and ligand-induced changes in the overall conformation analysed by scattering techniques; segmental deuteration of specific protein domains allowed for drastic reduction of the number of possible structures. Furthermore, this study illustrates how to evaluate and how to choose possible ligation junctions for sortagging that do not interfere with protein function.

An alternative enzyme-based ligation can be achieved with the Asx-specific (Asx = Asn or Asp) ligase family usually referred to as asparaginyl endoproteases (AEP).^128^ The most prominent member, butelase-1, was isolated from the tropical plant *Clitoria ternatea*.^129^ Butelase-1 requires only three amino acids (D/N-HV) for substrate recognition, provides a higher catalytic efficiency and has a broader sequence tolerance than Sortase A (Table 1).^130^ Many applications of butelase-1 ligation of synthetic and recombinant segments in protein synthesis and bioconjugation have been demonstrated.^131,132^ So far, butelase-1 has been applied mainly for peptide cyclization reactions; its application in ligation chemistry is restricted by the fact that it is not recombinantly available and must be isolated from plants.^132^ However, recent advances in protein engineering yielded a butelase-1-like ligase, which can be recombinantly expressed in *Sf9* insect cells.^133^ Another less active homolog of butelase-1 from *Oldenlandia affinis*, OaAEP, has been successfully produced in *E. coli*.^134^ Its catalytic efficiency is ∼90 times lower than butelase-1, but it has been used in ligations leading to site-specific^135^ or segmentally isotope labelled proteins such as maltose-binding protein (MBP), which were subsequently characterised by NMR spectroscopy.^136,137^ Advantages and disadvantages of enzymes used for protein ligation are summarised in Table 1.

Protein segments can be ligated using proteases in reverse, in addition to peptidyl transferases, which catalyse peptide bond formation through transacylation of amines. The reaction is based on engineered proteases such as subtiligase and trypsiligase. The latter enzyme is a trypsin variant capable of ligating two protein segments together if one segment carries an activated ester moiety at its C-terminus (Table 1). Efficient catalysis is dependent on a Y-RH recognition sequence and allows for introduction of modifications at either the N- or C-terminus of a protein under native conditions.^138^ For introduction of N-terminal PTMs, the released N-terminus of the RH-protein precursor can be conjugated with activated acyl donor substrate mimetics.^139^ Trypsiligase can also be exploited for the regioselective C-terminal modification of proteins through a transpeptidation reaction between a Y-RH recognition motif and a nucleophilic acyl moiety (Table 1). One major advantage of trypsiligase-mediated ligation reactions is the rapid reaction (usually within minutes). However, limitations for its use in protein semisynthesis are due to its high sequence specificity, which theoretically only allows traceless ligation of 0.5% of all known protein sequences, but suppresses undesired side reactions. Additionally, yields are lowered by hydrolysis and reversibility of the reaction.^130^

Subtiligase is a protease engineered for bioconjugation from the protease subtilisin isolated from *Bacillus amyloiquefaciens* (Table 1).^140^ Subtiligase variants have high catalytic activity and a broad sequence tolerance, but reversibility of the peptide bond formation remains a significant drawback.^141^ Subtiligases catalyse ligation between a peptide ester or thioester and the N-terminal α-amine of a protein or peptide, without the strict sequence specificity required by sortases, AEPs or trypsiligases. Ideally, the ligation site is flanked by hydrophobic amino acids (residues *P*_1_ and *P*_4_) at the N-terminal positions, whereas the C-terminal site is occupied by Arg, Met or small residues at *P*_1′_ and large, hydrophobic residues at *P*_2′_.^141^ Further engineering approaches have led to an enzyme called peptiligase, a Ca^2+^-independent enzyme for transpeptidation with higher stability than non-engineered subtiligase variants. Peptiligase has the ability to conjugate a C-terminal carboxyamidomethyl ester and a nucleophilic acyl acceptor without significant hydrolysis in an aqueous environment.^142^ Recent refinement attempts resulted in the generation of another subtiligase variant termed omniligase, which has a broad tolerance for substrates at the P_1′_ and P_2′_ positions.^143^ Together, subtiligase variants offer several advantages and a broad spectrum of possible applications (Table 1). For application in protein synthesis, substrates for the ligation reaction can be prepared *via* SPPS, which allows the site-specific incorporation of PTMs or other modifications. Alternatively, either or both substrates can be recombinantly expressed, allowing for isotope labelling. In comparison to native chemical ligation methodologies, enzyme-mediated ligations do not require a cysteine residue at the ligation site.^144,145^

In addition to ligating protein segments, enzyme-mediated protein conjugations can also be used to attach posttranslational modifications such as glycosylation or ubiquitination to a labelled protein. For instance, Slynko *et al.* employed a glycosyl transferase to attach a (unlabelled) glycan part enzymatically to the isotope labelled protein.^146^ This also applies to other PTMs such as acetylation or phosphorylation, where acetyltransferases^147^ or kinases^148^ can be employed for posttranslational modification of an isotope-labelled protein *in vitro.*

## Joining protein segments with non-native linkages

All (semi-)synthesis strategies described above are valuable tools for generating proteins from segments linked by native peptide bonds, which makes them indistinguishable from their native counterparts. In some cases, however, a non-native linkage between two protein segments might facilitate access to (modified) proteins or confer desired properties to the target protein (*e.g.* increased stability). To avoid undesired functional consequences caused by non-native connections, one needs to carefully select the site(s). Reactions used to create non-native linkages need to proceed quickly and be fully bioorthogonal, not affecting other functional groups in the protein of interest. Bio-orthogonal reactions typically take place on functional groups not found in proteins such as azide, alkyne, ketone or aldehyde moieties. Several excellent reviews of chemoselective and bioorthogonal reactions applied for protein modification and conjugation are available.^149–151^ Therefore, this review will remain focused on the use of such reactions in the context of specific isotope labelling.

**Fig. 7:**
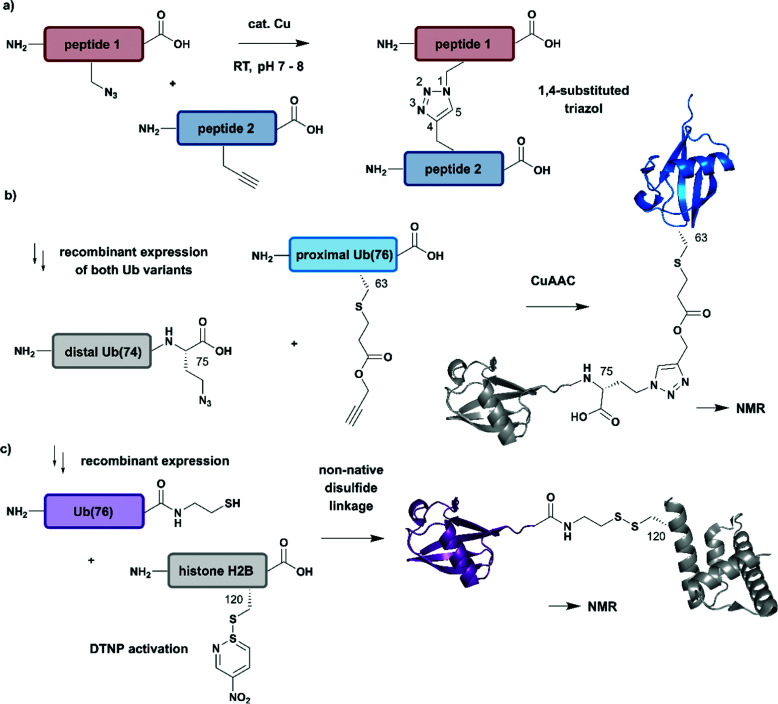
Non-native linkages for joining differently isotope–labelled protein segments. (a) General mechanism of copper(i)-catalyzed formation of a 1,4-substituted triazole linkage between two peptides (“click reaction, CuAAC”). (b) Segmental isotope labelling of the proximal Ub(76) subunit (cyan) by CuAAC. Both Ub segments were derived recombinantly (PDB: 1UBQ).^155^ (c) Site-specific conjugation of isotope-labelled Ub(76) to unlabelled histone H2B (both proteins were prepared recombinantly) by mimicking an isopeptide bond by a disulfide linkage. The obtained Ub-H2B complex analogue was characterised by NMR and revealed an acidic surface patch modulating its chromatin interaction (PDB: 6A7U).^46^ | Grey: unlabelled protein parts | cyan/purple: homogenous, stable isotope-labelled ubiquitin.

A popular strategy for bioconjugation is the copper(i)-catalyzed azide–alkyne cycloaddition (CuAAC), sometimes referred to as the “click reaction” (Fig. 7a).^152,153^ Both functionalities required for this reaction, azides and alkynes, can be easily incorporated into synthetic peptides or included in recombinant proteins using genetic code expansion tools to install non-proteinogenic amino acid building blocks *via* stop-codon suppression.^154^

The advantages of click-chemistry based approaches for the generation of artificially conjugated diUb were recently demonstrated by Schneider *et al.* (Fig. 7b).^155^ They generated artificially Lys11-, Lys27- or Lys63-linked diUb variants and assessed their conformational and functional properties by NMR spectroscopy. The unlabelled, distal Ub carried the unnatural amino acid l-azidohomoalanine in place of the C-terminal Gly75 and Gly76 residues. The labelled Ub variants, which bore single Lys-to-Cys mutations at the required positions, were reacted site-specifically with propargyl-acrylate and subsequently incubated with the distal Ub molecules. The following click CuAAC reaction resulted in an artificial triazole-linked diUb, which Schneider *et al.* used for the acquisition of NMR data, supported by molecular dynamics simulations. Although the derived molecules were artificially linked, their structural, dynamic and functional properties were assumed to mirror the behaviour of native isopeptide-linked Ub dimers. A comparison of the associated chemical shift perturbations by ^1^H–^15^N NMR correlation spectra between the triazole-linked variants^155^ and previously published isopeptide-linked diUb^85,156^ confirmed this hypothesis. The example described here nicely reflects the application of click chemistry to quickly link isotope labelled protein domains in a complex assembly. Segmental labelling increased the resolution of NMR signals of each individual Ub subunit, which would not have been possible by uniform labelling of the complete diUb due to multiple overlapping signals. However, the application of this labelling strategy is restricted to systems, such as that by Schneider *et al.*,^155^ in which a disadvantageous influence of the non-native linkage can be excluded.

Several strategies generating non-native linkages have also been applied for the conjugation of Ub to substrate proteins. For example, Ub attachment *via* disulfide linkages was described first by Chatterjee *et al.* and Chen *et al.* in 2010.^157,158^ This approach was further extended by Debelouchina *et al.* to site-specific conjugation of isotope-labelled Ub to unlabelled histone H2B.^159^ In this work, Ub was fused with an *Ava* DnaE intein and subsequently cleaved with cysteamine to generate Ub with a C-terminal thiol group (Fig. 7c). The single Cys residue (Lys120Cys) of the H2B mutant was activated with 2,2′-dithiobis(5-nitropyridine) (DTNP) after recombinant expression. Co-incubation of H2B- with Ub bearing a C-terminal thiol yielded a disulfide analogue of Ub-H2B, which then was incorporated into nucleosomes. By using a ^1^H–^2^H exchange strategy in combination with NMR spectroscopy, Debelouchina *et al.* characterised the effects of ubiquitylation on chromatin modulation and structure. These experiments revealed an acidic surface patch comprising two Glu residues on Ub, which are essential for decompaction of the nucleosome. This strategy significantly accelerated the generation of protein variants carrying isotope labelled ubiquitin, however the instability of the introduced disulfide linkage to reducing conditions should be noted.^159^

## Beyond the scope of segmental and site-specific isotope labelling

**Fig. 8:**
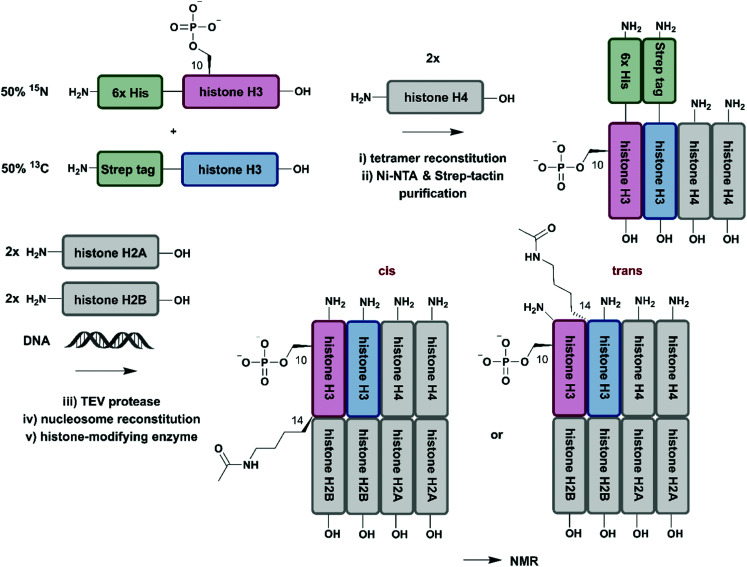
Isotope labelling of individual subunits in a protein complex. All protein segments were prepared by recombinant expression. A sequential Ni–NTA and Strep-tactin separation workflow yielded asymmetrically phosphorylated and ^13^C/^15^N (blue/red) labelled H3/H4 tetramers, which were employed for reconstitution of functional nucleosomes by addition of unlabelled H2A, H2B and DNA. Upon incubation of reconstituted nucleosomes with acetyltransferase Gcn5, PTM-induced crosstalk in *cis* and in *trans* could be studied by time-resolved NMR spectroscopy.^161^ | Grey: unlabelled protein segments | red: homogenous, ^15^N stable isotope-labelled protein segment | blue: homogenous, ^13^C stable isotope-labelled protein segment | green: affinity purification tags.

So far, we have discussed several methodologies for incorporation of isotope labeling patterns into segments of proteins. Alternative strategies, such as the chemical modification of lysine sidechains by ^13^C-methylation,^160^ can introduce stable isotopes into proteins generated by conventional methods and they can deliver valuable insight into protein–protein interactions without the need to deal with elaborate expression conditions and/or bioconjugation approaches. Furthermore, a discussion of segmental labelling would be incomplete without mentioning approaches that address multi-protein complexes and methods to control their assembly with individually labelled protein components. Incorporation of stable isotopes into a single protein in a larger complex can deliver valuable insights into its role and behavior in a complex environment. A prime example of protein-specific isotope labeling in a larger complex has been provided by Liokatis *et al.*^161^ They focussed on differential isotope-labelling and site-selective incorporation of PTMs (acetylation and phosphorylation) in an octameric histone complex (Fig. 8). For their experiment, the authors recombinantly expressed histone H3 variants in M9 minimal media containing ^15^NH_4_Cl or ^13^C-glucose either fused to a 6xHis-tag (for ^15^N-labelled H3) or a Strep-tag (for ^13^C-labelled H3), which allowed for purification by a two-step affinity process of all histone H3 pools after enzymatic phosphorylation and reconstitution with histone H4. Afterwards, the phosphorylated and purified H3/H4 tetramers were incubated with unlabelled histone H2A and H2B and DNA, resulting in fully reconstituted, asymmetrically modified nucleosomes (asNucs). In order to assess the reciprocal PTM crosstalk between acetylation and phosphorylation in these segmentally labelled asNucs, they were incubated with the acetyltransferase Gcn5. Due to the sophisticated labelling approach it was possible to follow the acetylation of H3K14 in a time-resolved manner on both H3 tails independently. The recorded ^1^H–^15^N and ^1^H–^13^C NMR spectra displayed a stimulatory effect of Ser10 phosphorylation. This influence on histone modification was described previously by the same group,^162^ on the acetylation of Lys14 in *cis*, whereas the reaction was delayed in *trans*. The procedure was further exploited in experiments for studying PTM crosstalk between Lys methylation and Thr phosphorylation. Liokatis *et al.* incubated the asNucs in a first step with a Lys methyltransferase, an enzyme whose activity can be monitored in a time-resolved manner by NMR,^163^ and then monitored the H3 Thr3 phosphorylation by NMR. These experiments revealed an inhibitory effect of monomethylated Lys residues on the phosphorylation activity of the Haspin kinase.^161^ The approach described by Liokatis *et al.* offers an opportunity to specifically detect and monitor enzymatic PTM changes of individual histones in an nucleosome assembly and can be interfaced with other controlled assembly routes for nucleosomes,^164^ and to study the dynamic nucleosome landscape.^165^

## Conclusions

The methodologies and examples reviewed here demonstrate strategies for segmental and site-specific labelling of proteins, facilitating their detailed characterisation by spectroscopic applications. The application of NCL (or alternative ligation reactions) to stitch together synthetic peptides allows for precise incorporation of labelled amino acids as well as other modifications such as PTMs. However, high costs and limited availability of building blocks together with size limitations of synthetic peptides restrict its broader use in the field of isotope labelling – a drawback that might be overcome by the development of cost-effective methods to generate isotope labelled amino acids suitably protected for solid phase peptide synthesis.^62,166^ EPL circumvents these challenges by accessing isotope labelled protein segments using recombinant expression, which also removes size limitations. In combination with SPPS, posttranslational modifications can be incorporated into segmentally labelled proteins. Such semisynthetic proteins offer the possibility for detailed structural and functional characterisation, even allowing for monitoring of weak and dynamic interactions.^167^ A major disadvantage, however is that the synthetic segment bearing the PTMs is typically unlabelled and therefore short-range structural changes caused by the PTM(s) are not observed. EPL also suffers from drawbacks such as limitations based on sequence and protein properties that might prevent expression at the required scale and/or need time-consuming optimization of workflows, which often require extensive expertise in expression and peptide synthesis not readily available in many laboratories.

Similar challenges apply to segmental isotope labelling and site-specific modification of proteins by PTS, with respect to engineering of split intein fusion constructs. However, PTS reactions can be achieved at relatively low concentrations and under physiological conditions, which has made PTS one of the most used approaches to segmentally labelled proteins.^56^ Similarly popular are some enzyme-mediated ligation approaches, *e.g.* based on Sortase A, which offer a rapid and easy workflows for segmental labelling.^130^ However, the resulting ligation products often carry a ligation “scar” comprising non-native amino acids at the ligation site (with the exception of subtiligase). Non-native linkages, even though more subtle, also result from the use of biorthogonal ligations such as CuAAC to generate modified proteins.

In summary, the methods discussed here are of significant value to the protein chemistry and spectroscopy communities when zooming into complex protein structures by using selective isotope labelling strategies to study (dynamic) effects of PTMs on specific residues or domains. We expect that their relevance will further increase as protein chemistry and protein engineering approaches become more easily accessible to biochemistry and structural biology groups. However, for now the few examples that document the power of selective isotope labelling to study the effects of PTMs on proteins are clear evidence for the fact that more intensive collaborations between protein chemists and structural biologists are needed as both aspects require high levels of expertise and are seldom combined. Continuing developments in protein (semi-)synthesis, such as the diselenide-selenoester ligation (DSL)^168^ or the use of solubilization tags,^169^ now allow protein assembly at low concentrations, at variable sites and of hydrophobic peptide segments. These advances are complemented by newly emerging flexible and traceless enzymatic ligation reactions as well as by better access to isotope labelled amino acids and metabolic precursors. Together with spectroscopic advances that allow higher-resolution data to be obtained with smaller amounts of proteins, such as dynamic nuclear polarization (DNP) NMR^170^ or dissolution DNP methods^171^ that can also be employed in native environments such as cells,^172^ boundaries with respect to the complexity of systems analyzed will be pushed.

## Conflicts of interest

There are no conflicts to declare.

## Supplementary Material
